# Pain and disability following first-time lumbar fusion surgery for degenerative disorders: a systematic review protocol

**DOI:** 10.1186/s13643-016-0252-2

**Published:** 2016-05-03

**Authors:** Niek Koenders, Alison Rushton, Nicola Heneghan, Martin L. Verra, Paul Willems, Thomas Hoogeboom, J Bart Staal

**Affiliations:** Department of Physiotherapy, Radboud Institute for Health Sciences, Radboud University Medical Centre, Nijmegen, The Netherlands; School of Sport, Exercise and Rehabilitation Sciences, College of Life and Environmental Sciences, University of Birmingham, Edgbaston, Birmingham, B15 2TT UK; Department of Physiotherapy, Bern University Hospital, Bern, Switzerland; Maastricht University Medical Centre, Maastricht, The Netherlands; Radboud University Medical Centre, Radboud Institute for Health Sciences, IQ Healthcare, Nijmegen, The Netherlands; Research Group Musculoskeletal Rehabilitation, HAN University of Applied Sciences, Nijmegen, The Netherlands

**Keywords:** Spinal fusion, Spinal surgery, Pain, Disability, Spinal stenosis, Spondylolisthesis, Herniated disc

## Abstract

**Background:**

Lumbar spinal fusion for degenerative disorders of the lumbar spine is frequently used, despite current research presenting inconclusive evidence. This study aims to systematically review and meta-analyse the natural course of pain and disability in patients with degenerative disorders of the lumbar spine such as spinal stenosis, spondylolisthesis, disc herniation, or discogenic low back pain to improve lumbar spinal fusion management.

**Methods/design:**

An electronic database search will be conducted up to 30 September 2015 using MEDLINE, EMBASE, CINAHL, and ZETOC database. In addition, a search for articles in press and published ahead of print, British National Bibliography for Report Literature, and OpenGrey will be conducted. Prospective cohort studies using outcome measures of pain and disability will be eligible for inclusion. Two reviewers will screen titles, abstracts, and full-text independently using predetermined inclusion and exclusion criteria. The risk of bias of included studies will be assessed with the modified version of the Quality in Prognostic Studies tool. If meta-analysis of outcome data is deemed appropriate, variance-weighted pooled means will be calculated.

**Discussion:**

The results of this systematic review and meta-analysis may improve understanding of recovery after lumbar spinal fusion and improve lumbar spinal fusion management.

**Systematic review registration:**

PROSPERO CRD42015026922

**Electronic supplementary material:**

The online version of this article (doi:10.1186/s13643-016-0252-2) contains supplementary material, which is available to authorized users.

## Background

Lumbar spinal fusion (LSF) is a surgical procedure which aims to decompress and stabilize the lumbar spine in various degenerative disorders such as spinal stenosis, spondylolisthesis, disc herniation, and discogenic low back pain [1–3]. Data provided by the US Department of Health and Human Services shows a substantial increase in hospitalizations for spinal fusion in the USA from 61,000 in 1993 to 296,211 in 2002 and over 451,000 in 2012 [4]. Similarly, the contribution of spinal fusion to the national bill in the USA increased from $4.3 billion to $33.9 billion between 1998 and 2008 [5]. Ageing and surgical advancement are likely to contribute to a further raise in use of LSF [6].

The increasing use of LSF is remarkable, since definite proof of treatment efficacy of LSF for symptomatic degenerative lumbar spine conditions is still lacking [7]. For example, there is insufficient evidence from randomized controlled trials supporting positive outcomes after surgery compared to nonsurgical treatment in patients with degenerative lumbar spondylolysis [8]. Furthermore, Atlas et al. [9] report in their prospective cohort study that long-term low back pain and patient satisfaction are similar regardless of surgical or nonsurgical treatment in patients with lumbar spinal stenosis. Pekkanen et al. [10] show in their prospective cohort study a decrease in disability after LSF for degenerative conditions, although the patients did not reach similar disability outcomes compared to a general population at 1-year follow-up. In addition, several studies analysing cost-effectiveness report questionable outcomes of LSF in patients with degenerative spondylolisthesis [11–13]. Moreover, LSF is not without any risks given the incidence of graft-specific complications (5.4–10.0 % [14–16]) and revisions (2.0–6.9 % [17–21]). Phillips et al. [22] report in their systematic review that LSF compared to nonsurgical treatment significantly decreases pain and disability in patients with refractory chronic low back pain. However, the methodology of this study is criticized because of non-reporting of methodological quality of included studies, an unclear selection of studies, and inadequate pooling of results [23]. Finally, the positive effect of LSF on patients with chronic low back pain seems to decrease at longer follow-up [24]. Therefore, LSF might not be effective for the entire heterogeneous group of patients [25].

In summary, LSF is increasingly used as treatment of degenerative disorders of the lumbar spine while evidence seems to show inconclusive outcomes and questionable cost-effectiveness. In particular, there is lack of understanding of long-term outcomes after LSF [8]. An overview of the natural course of pain and disability in current LSF management is needed to improve understanding of recovery after LSF and to gain insight into optimal timing of rehabilitation or physiotherapy in the period after LSF. To the knowledge of the authors, no overview of the natural course after LSF exists. Therefore, the main objective is to systematically review and meta-analyse the natural course of pain and disability in patients with degenerative disorders of the lumbar spine such as spinal stenosis, spondylolisthesis, disc herniation, or discogenic low back pain after first-time LSF surgery.

## Methods/design

### Search strategy

A comprehensive electronic search will be conducted in MEDLINE, EMBASE, CINAHL, and ZETOC database to 30 September 2015 (Additional file 1). In addition, a search for articles in press and published ahead of print will be conducted in relevant journals for spine surgery (e.g. Spine; The Spine Journal; European Spine Journal; Journal of Neurosurgery: Spine; International Journal of Spine Surgery; Global Spine Journal) and reference lists of included studies will be searched for further relevant studies. Furthermore, a search in the British National Bibliography for Report Literature and OpenGrey will be performed to identify unpublished studies. An experienced medical librarian was consulted in designing the search strategy. The language of publication will not be restricted.

### Selection of studies and eligibility criteria

Titles and abstracts (stage 1) followed by full-texts of potentially relevant studies (stage 2) will be independently screened by two reviewers (NK and TH). Eligibility of the study will be graded as eligible, not eligible, or might be eligible [26] using the eligibility criteria presented in Additional file 2: Table S1. Where no abstract is available, full-text articles will be obtained unless the article can be confidently excluded by its title alone. In general, if there is any doubt about exclusion of the study, the study will proceed to the full-text screening stage to reduce the likelihood of excluding a relevant study. Disagreements will be solved by consensus. Where no consensus can be reached, a third party (AR) will arbitrate [27]. The process of study selection will be summarized using a Preferred Reporting Items for Systematic Reviews and Meta-Analyses (PRISMA) flow diagram [28].

### Data extraction and management

Data for each included study will be extracted using a standardized form managed in Microsoft Access (Microsoft Corporation, Seattle, WA, USA). Prior to data extraction, piloting of the form will be conducted in a small number of studies (e.g. ≤5). Data extraction will be performed independently and in duplicate.

Data extracted for each study will include the following summary data: participants (setting and area), patient characteristics, duration of symptoms, outcomes (including scale and name of the questionnaire/instrument), surgical procedure, clinical care pathway, design, sample size, inclusion and exclusion criteria, and follow-up dates. In addition, data will be collected regarding possible determinants for effect modification (Additional file 3: Table S2).

### Outcome measures

Results considering pain or disability will be reported for the entire population and per patient category (spinal stenosis, spondylolisthesis, disc herniation, discogenic low back pain). Data from studies without a detailed description of outcomes per patient category will be presented in the category “blended”. Pain and disability outcome measures are primary outcomes and will be measured with, for example, Visual Analogue Scale (VAS), Numeric Rating Scale (NRS), Oswestry Disability Index (ODI), Roland Disability Questionnaire (RDQ), or Quebec Back Pain Disability Questionnaire (QBPDQ). The outcome data will be presented at an original scale or converted to a 0–100 scale if appropriate [29].

### Assessment of risk of bias of included studies

Risk of bias for each included study will be independently assessed by the same initial reviewers (NK and TH); the third reviewer (AR) will mediate in situations of disagreement. Cohen’s *κ* will be used to assess agreement between the reviewers. All tools and processes will be piloted prior to use. Risk of bias will be assessed using the modified version of the Quality in Prognostic Studies (QUIPs) tool, originally developed by Hayden et al. [30]. Studies will be assessed based on the domains of representation of sample, definition of study sample, study attrition, outcome measurement, confounding, statistical analysis, provision of data, and blinding of outcomes (modified version: Additional file 4).

### Dealing with missing data

In case of missing data, authors will be contacted to provide additional information. If missing values (i.e. mean and variance) cannot be retrieved, the formula of Hozo et al. [31] will be used to estimate mean and variance with use of median, range, and sample size. Headrick’s formula [32] will be used to combine means when separate means describe results of one study group.

### Assessment of heterogeneity

The statistical heterogeneity will be analysed using the *I*^2^ [33]. The literature suggests 25 % as low heterogeneity, 50 % as moderate, and 75 % as high [33].

### Assessment of reporting biases

**Fig. 1:**
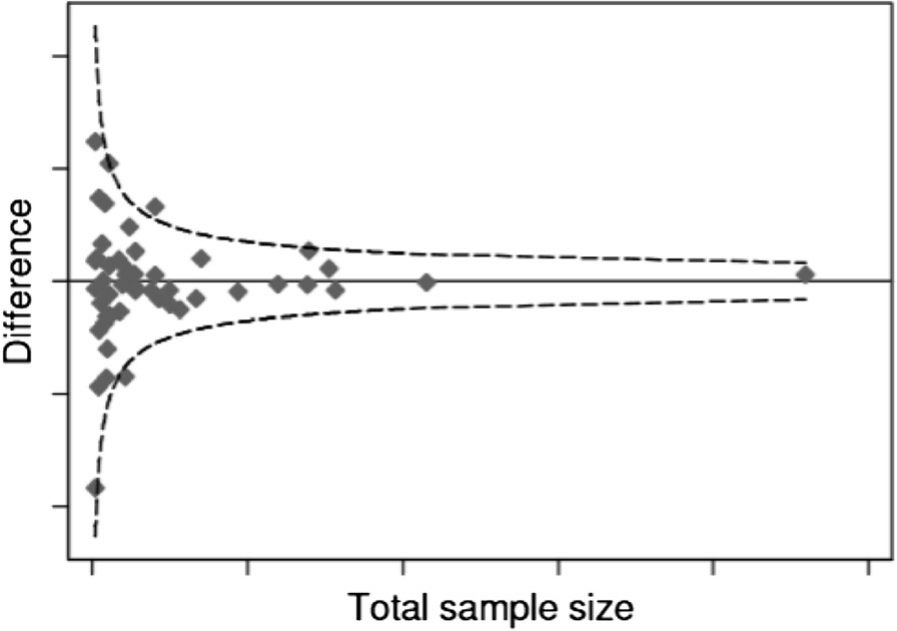
Example of a modified funnel plot; outcome versus total sample size

To assess location bias and outcome reporting bias [27], relevant study characteristics such as the indexing of studies in electronic databases and reported outcome measures will be described. If there are sufficient numbers of studies available (i.e. ≥10), a modified funnel plot (Fig. 1) will be constructed to assess for possible publication bias. A modification of a standard funnel plot [27] is needed to assess selective publication of change on pain and disability outcomes in relation to the study sample size.

### Data synthesis

If possible, a meta-analysis will be conducted on pain and/or disability outcome data with the use of Stata and R [34, 35]. Variance-weighted pooled estimates of outcomes will be calculated for the continuous data [29]. Minimal important change values as provided by Ostelo et al. [36] (VAS 15, NRS 2, ODI 10, RDQ 5, QBPDQ 20) will be used to interpret results and draw conclusions regarding a satisfying or disappointing natural course of pain and disability after LSF. However, it needs to be stressed that these values are for individual rather than group changes. Therefore, the method of Guyatt et al. [37] will be applied to estimate proportions of patients who benefit from treatment.

### Reporting of the review

The results will be reported in accordance with the PRISMA statement and its checklist [28]. A completed copy of the PRISMA checklist will be provided in the additional files (Additional file 5) (PROSPERO CRD42015026922).

## Discussion

This systematic review and meta-analysis will provide an overview of the natural course of pain and disability in patients with degenerative disorders of the lumbar spine after first-time LSF surgery. The results could provide valuable information what would improve our understanding of recovery after LSF and serve as a rigid foundation for comparison of LSF outcomes of future studies. Ultimately, the results may lead to changes in timing of adequate LSF management and decision making for both patients and surgeons.

In this systematic review and meta-analysis, it is necessary to anticipate on a few challenges. First, there could be a high heterogeneity in used surgical procedures (e.g. open versus minimally invasive) as a result of lack of evidence regarding safety and efficacy of different procedures [38]. Where possible, data will be pooled and analysed within the same surgical procedure. Secondly, it is possible that the continuous outcome data on the same construct needs to be converted to a 0 to 100 scale or percentage to increase comparability of data between studies [36]. Percentages could improve the ability to interpret change between outcome measures [36].

## Additional files

Additional file 1: Search strategy example. Description of data: The data provided shows an example of a comprehensive electronic search conducted in MEDLINE. (DOCX 27 kb) Additional file 2: Table S1. Population, intervention, comparator, outcomes, study design, and time breakdown of study eligibility criteria (DOCX 28 kb) Additional file 3: Table S2. Possible determinants for effect modification (DOCX 37 kb) Additional file 4: Modified QUIPs tool. The data presents the modified version of the Quality in Prognostic Studies (QUIPs) tool which will be used to assess risk of bias of included studies. (DOCX 25 kb) Additional file 5: Preferred Reporting Items for Systematic Reviews and Meta-analysis Protocols checklist. The data shows a completed copy of the PRISMA checklist to guide readers in assessment of the quality of the current review protocol article. (DOCX 26 kb)

## Abbreviations

LSF: lumbar spinal fusion
PRISMA: Preferred Reporting Items for Systematic Reviews and Meta-Analyses
QUIPs: Quality in Prognostic Studies
