# Supplementary material for: Pain and disability following first-time lumbar fusion surgery for degenerative disorders: a systematic review protocol
Source: Syst Rev. 2016 May 3;5:72. doi: 10.1186/s13643-016-0252-2 (PMC4855758; doi:10.1186/s13643-016-0252-2)
Supplement: Additional file 1: — Search strategy example. Description of data: The data provided shows an example of a comprehensive electronic search conducted in MEDLINE. (DOCX 27 kb) [file 13643_2016_252_MOESM1_ESM.docx]

*Additional file 1; Search strategy example*

**MEDLINE**

### Patient-intervention terms

1. Spinal Fusion/
2. ((Spin* adj3 fusion?) or (fusion? adj3 spin*) or (spondylosyndes?s) or (spondylodes?s) or ((spin* or vertebra?) adj3 arthrodes?s)).ti,ab,kf.
3. ((lumbar or posterolateral or interbody or anterior or posterior or transforam?nal or transpsoas or facet or pedicle or cage or verterbra? or oblique) adj3 fusion?).ti,ab,kf.
4. ((minim* invasive adj5 fusion?) or (mini-open adj5 fusion?)).ti,ab,kf.

### Outcome terms

1. ((Natural history) or ((natural or clinical) adj3 course)).ti,ab,kf.
2. pain/ or back pain/ or low back pain/ or neuralgia/ or sciatica/ or pain, referred/ or pain, postoperative/ or treatment outcome/
3. (pain? or ache? or ((backache? lower) or (back ache? lower) or (back pain? lower) or (backpain? lower)) or ((backache? low) or (back ache? low) or (back pain? low) or (backpain? low)) or ((ache? lower back) or (pain? lower back)) or ((ache? low back) or (pain? low back)) or ((lower backache?) or (lower back ache?) or (lower back pain?) or (lower backpain?)) or ((low backache?) or (low back ache?) or (low back pain?) or (low backpain?)) or lumbago or ((pain? radiating) or (radiating pain?)) or (neuralgia? or neuralgic?) or ((nerve pains?) or (pain? nerve)) or ((neuropathic pain?) or (pain? neuropathic)) or sciatica or ((neuralgi? sciatic?) or (sciatic? neuralgi?)) or ((referred adj3 pain) or (pain adj3 referred)) or ((postoperative adj3 pain?) or (pain adj3 postoperative))).ti,ab,kf.
4. activities of daily living/ or disability evaluation/
5. ((daily living activit*) or (activit* daily living) or (activit* of daily living) or (living activit* daily) or adl or (disability evaluation?) or (evaluation? disability) or outcome).ti,ab,kf.
6. pain measurement/ or pain perception/ or visual analog scale/
7. ((pain assessment) or (pain scale) or (McGill pain questionnaire) or MPQ or (SF-MPQ) or (numeric rating scale) or NRS or (NRS-11) or (NRS-101) or (numeric rating score) or (visual analogue scale) or VAS or (visual analogue score) or (visual analog* scale) or (analog* visual scale) or (scale visual analog*)).ti,ab,kf.
8. (questionnair* or (Oswestry adj5 disability index) or ODI or mODI or OSW or mOSW or (Oswestry adj5 disability questionnaire) or ODQ or (Quebec adj5 disability scale) or QBPDS or QUE or QDS or (Roland adj5 disability questionnaire) or (Roland-Morris adj5 disability questionnaire) or (Roland-Morris adj5 disability questionnaire) or RDQ or RDQ-11 or RDQ-18 or RDQ-25 or M-RDQ or mRDQ or RMDQ or RMDQ-11 or RMDQ-18 or RMDQ-24 or M-RMDQ or mRMDQ).ti,ab,kf.
9. exercise test/ or walking/
10. ((physical performance assessment?) or (walk test?) or (walk-test?) or (walk distance) or 6MWT or 6MWTD or 2MWT or 5MWT or 12MWT or 15MWT or 10MWT or 10MTWT or FWT or 10MSWT or SPWT or (timed adj5 test) or TUG or (performance oriented mobility assessment) or POMA or (elderly mobility scale) or EMS).ti,ab,kf.

### Study terms

1. epidemiologic studies/ or exp case control studies/ or exp cohort studies/ or (case control).tw. or (cohort adj (study or studies)).tw. or (cohort analy$).tw. or (follow up adj (study or studies)).tw. or (observational adj (study or studies)).tw. or longitudinal.tw. or prospective.tw.
