# Supplementary material for: Pain and disability following first-time lumbar fusion surgery for degenerative disorders: a systematic review protocol
Source: Syst Rev. 2016 May 3;5:72. doi: 10.1186/s13643-016-0252-2 (PMC4855758; doi:10.1186/s13643-016-0252-2)
Supplement: Additional file 2: Table S1. — Population, intervention, comparator, outcomes, study design, and time breakdown of study eligibility criteria (DOCX 28 kb) [file 13643_2016_252_MOESM2_ESM.docx]

**Table 1;** Population, intervention, comparator, outcomes, study design, and time breakdown of study eligibility criteria

| **Category** | **Description of criteria** |
| --- | --- |
| Population | Adult patients (aged over 16 years) with degenerative disorders of the lumbar spine such as spinal stenosis (including spondylolisthesis)([Kreiner et al., 2013](#_ENREF_2)), disc herniation([Kreiner et al., 2014](#_ENREF_1)), or discogenic low back pain who have undergone first time lumbar spinal fusion surgery. |
| Intervention | Lumbar spinal fusion, both open or minimally invasive surgery. |
| Comparator | None. |
| Outcomes | Primary outcomes   - Pain (e.g. Visual Analogue Scale or Numeric Rating Scale) - Disability (e.g. Roland Disability Questionnaire, Oswestry Disability Index or Quebec Back Pain Disability Questionnaire)   Secondary outcomes   - Work absenteeism - Quality of life - Adverse events - Health service utilisation |
| Study design | Prospective cohort studies with consecutive patient sampling. |
| Time | Outcomes will be collected for short term (≤ 3 months follow-up), medium term (> 3 months, ≤ 12 months), and long term (> 12 months). Long-term outcomes are considered outcomes of main interest. |

**References**

Kreiner, D. S., Hwang, S. W., Easa, J. E., Resnick, D. K., Baisden, J. L., Bess, S., . . . Toton, J. F. (2014). An evidence-based clinical guideline for the diagnosis and treatment of lumbar disc herniation with radiculopathy. *Spine J, 14*(1), 180-191. doi: 10.1016/j.spinee.2013.08.003

Kreiner, D. S., Shaffer, W. O., Baisden, J. L., Gilbert, T. J., Summers, J. T., Toton, J. F., . . . Reitman, C. A. (2013). An evidence-based clinical guideline for the diagnosis and treatment of degenerative lumbar spinal stenosis (update). *Spine J, 13*(7), 734-743. doi: 10.1016/j.spinee.2012.11.059
