# Supplementary material for: Pain and disability following first-time lumbar fusion surgery for degenerative disorders: a systematic review protocol
Source: Syst Rev. 2016 May 3;5:72. doi: 10.1186/s13643-016-0252-2 (PMC4855758; doi:10.1186/s13643-016-0252-2)
Supplement: Additional file 3: Table S2. — Possible determinants for effect modification (DOCX 37 kb) [file 13643_2016_252_MOESM3_ESM.docx]

**Table 2;** Possible determinants for effect modification

| **Determinant** | **Study** | **Expected influence** |
| --- | --- | --- |
| Open or minimally invasive surgery | ([Vertuani et al., 2015](#_ENREF_7)) | Open surgery is predictive of lower outcomes after surgery |
| Levels affected | ([Turner et al., 1992](#_ENREF_6)) | Two or three-level fusion is predictive of lower outcomes after surgery |
| Severity of complaints before surgery | ([Abbott, Tyni-Lenne, & Hedlund, 2011](#_ENREF_1)) | High leg pain intensity is predictive of high leg pain after surgery |
| Duration of symptoms before surgery | ([Marshman, Kasis, Krishna, & Bhatia, 2010](#_ENREF_5)) | No influence |
| Disability before surgery | Rushton et al., Prognostic factors for lumbar spine fusion surgery, Submitted | Lower disability before surgery is predictive of lower disability at 12-months after surgery |
| Age | Rushton et al., Prognostic factors for lumbar spine fusion surgery, Submitted | No influence |
| Gender | Rushton et al., Prognostic factors for lumbar spine fusion surgery, Submitted | No influence |
| Smoking before surgery | ([Wilson-MacDonald et al., 2008](#_ENREF_9)) | Current smoking is predictive of higher leg pain after surgery |
| Obesity | ([Lingutla et al., 2015](#_ENREF_4)) | No influence |
| Work status | ([Katz, 2006](#_ENREF_3)) | Psychologically stressful work is predictive of lower outcomes after surgery |
|  | ([Wilson-MacDonald et al., 2008](#_ENREF_9)) | Currently not in paid employment is predictive of lower outcomes after surgery |
|  | ([DeBerard, Masters, Colledge, Schleusener, & Schlegel, 2001](#_ENREF_2)) | Low household income is predictive of lower outcomes after surgery |
| Pain catastrophizing | ([Abbott et al., 2011](#_ENREF_1)) | Pain-related negative thinking is predictive of higher pain intensity after surgery |
| Depression before surgery | ([Wilhelm et al., 2015](#_ENREF_8)) | Depression present and/or high scores on depression scales is predictive of lower outcomes after surgery |

**References**

Abbott, A. D., Tyni-Lenne, R., & Hedlund, R. (2011). Leg pain and psychological variables predict outcome 2-3 years after lumbar fusion surgery. *Eur Spine J, 20*(10), 1626-1634. doi: 10.1007/s00586-011-1709-6

DeBerard, M. S., Masters, K. S., Colledge, A. L., Schleusener, R. L., & Schlegel, J. D. (2001). Outcomes of posterolateral lumbar fusion in Utah patients receiving workers' compensation: a retrospective cohort study. *Spine (Phila Pa 1976), 26*(7), 738-746; discussion 747.

Katz, J. N. (2006). Lumbar disc disorders and low-back pain: socioeconomic factors and consequences. *J Bone Joint Surg Am, 88 Suppl 2*, 21-24. doi: 10.2106/jbjs.e.01273

Lingutla, K. K., Pollock, R., Benomran, E., Purushothaman, B., Kasis, A., Bhatia, C. K., . . . Friesem, T. (2015). Outcome of lumbar spinal fusion surgery in obese patients: a systematic review and meta-analysis. *Bone Joint J, 97-B*(10), 1395-1404. doi: 10.1302/0301-620x.97b10.35724

Marshman, L. A., Kasis, A., Krishna, M., & Bhatia, C. K. (2010). Does symptom duration correlate negatively with outcome after posterior lumbar interbody fusion for chronic low back pain? *Spine (Phila Pa 1976), 35*(6), 657-665.

Turner, J. A., Ersek, M., Herron, L., Haselkorn, J., Kent, D., Ciol, M. A., & Deyo, R. (1992). Patient outcomes after lumbar spinal fusions. *JAMA, 268*(7), 907-911.

Vertuani, S., Nilsson, J., Borgman, B., Buseghin, G., Leonard, C., Assietti, R., & Quraishi, N. A. (2015). A Cost-Effectiveness Analysis of Minimally Invasive versus Open Surgery Techniques for Lumbar Spinal Fusion in Italy and the United Kingdom. *Value Health, 18*(6), 810-816. doi: 10.1016/j.jval.2015.05.002

Wilhelm, M., Reiman, M., Goode, A., Richardson, W., Brown, C., Vaughn, D., & Cook, C. (2015). Psychological Predictors of Outcomes with Lumbar Spinal Fusion: A Systematic Literature Review. *Physiother Res Int*. doi: 10.1002/pri.1648

Wilson-MacDonald, J., Fairbank, J., Frost, H., Yu, L. M., Barker, K., Collins, R., & Campbell, H. (2008). The MRC spine stabilization trial: surgical methods, outcomes, costs, and complications of surgical stabilization. *Spine (Phila Pa 1976), 33*(21), 2334-2340. doi: 10.1097/BRS.0b013e318186a8b2
