# Supplementary material for: Pain and disability following first-time lumbar fusion surgery for degenerative disorders: a systematic review protocol
Source: Syst Rev. 2016 May 3;5:72. doi: 10.1186/s13643-016-0252-2 (PMC4855758; doi:10.1186/s13643-016-0252-2)
Supplement: Additional file 4: — Modified QUIPs tool. The data presents the modified version of the Quality in Prognostic Studies (QUIPs) tool which will be used to assess risk of bias of included studies. (DOCX 25 kb) [file 13643_2016_252_MOESM4_ESM.docx]

*Additional file 2; Modified QUIPs tool*

Risk of bias assessment tool, modified with use of QUIPs tool ([Hayden, van der Windt, Cartwright, Cote, & Bombardier, 2013](#_ENREF_1)) and Pengel et al. ([Pengel, Herbert, Maher, & Refshauge, 2003](#_ENREF_2)).

| **Study participation (**[**Hayden et al., 2013**](#_ENREF_1)**)**  **Representative sample  (Pengel et al. 2003)** | **Defined sample**  **(Pengel et al. 2003)** | **Study attrition / complete follow up**  **(**[**Hayden et al., 2013**](#_ENREF_1)**)** | **Outcome measurement**  **(**[**Hayden et al., 2013**](#_ENREF_1)**)** | **Study confounding**  **(**[**Hayden et al., 2013**](#_ENREF_1)**)** | **Statistical analysis and reporting**  **(**[**Hayden et al., 2013**](#_ENREF_1)**)** | **Provision of data**  **(**[**Hayden et al., 2013**](#_ENREF_1)**)** | **Blinded outcome**  **(Pengel et al. 2003)** | **Overall statement of risk of bias** |
| --- | --- | --- | --- | --- | --- | --- | --- | --- |
| Data related to outcome may be different for participants and eligible non-participants /  Participants selected by random selection or as consecutive cases | Description of source of participants and inclusion and exclusion criteria | Data related to outcome may be different for completing and non-completing participants | Measurement of the outcome may be different related to the baseline level | Outcome may be distorted by another factor related to outcome | Reported results may be spurious or biased related to analysis or reporting | Studies must provide raw data, percentages, or continuous outcomes | Assessor blinded and unaware of other measures at time of outcome was measured | Number of low, moderate and high ratings |

*Note: Prognostic factor section of QUIPS not relevant.
QUIPS: QUality In Prognostic Studies;*

Hayden, J. A., van der Windt, D. A., Cartwright, J. L., Cote, P., & Bombardier, C. (2013). Assessing bias in studies of prognostic factors. *Ann Intern Med, 158*(4), 280-286. doi: 10.7326/0003-4819-158-4-201302190-00009

Pengel, L. H., Herbert, R. D., Maher, C. G., & Refshauge, K. M. (2003). Acute low back pain: systematic review of its prognosis. *BMJ, 327*(7410), 323. doi: 10.1136/bmj.327.7410.323
